# Gut microbiota in treating inflammatory digestive diseases: Current challenges and therapeutic opportunities

**DOI:** 10.1002/imt2.265

**Published:** 2024-12-31

**Authors:** Yongpeng Shi, Zeran Chen, Tingyu Fang, Xingyao Chen, Youpeng Deng, Hao Qin, Min Lian, Juntao Shen, Yuru Zong, Huikuan Chu, Constanze Hoebinger, Hao Guo, Zhongshang Yuan, Jie Zheng, Yongjian Zhou, Yue Pan, Beatriz G. Mendes, Sonja Lang, Tim Hendrikx, Suling Zeng, Hailong Cao, Ling Yang, Lianmin Chen, Peng Chen, Lei Dai, Hua Wang, Shi Yin, Shu Zhu, Xiong Ma, Bernd Schnabl, Hanqing Chen, Yi Duan

**Affiliations:** ^1^ Department of Infectious Diseases, The First Affiliated Hospital of USTC, Division of Life Sciences and Medicine University of Science and Technology of China Hefei China; ^2^ Center for Advanced Interdisciplinary Science and Biomedicine of IHM, Division of Life Sciences and Medicine University of Science and Technology of China Hefei China; ^3^ Key Laboratory of Immune Response and Immunotherapy, Division of Life Sciences and Medicine University of Science and Technology of China Hefei China; ^4^ Department of Nutrition and Food Hygiene, School of Public Health Capital Medical University Beijing China; ^5^ CAS Key Laboratory for Biomedical Effects of Nanomaterials and Nanosafety, CAS Center of Excellence in Nanoscience, National Center for Nanoscience and Technology Beijing China; ^6^ Division of Gastroenterology and Hepatology, Key Laboratory of Gastroenterology and Hepatology, Ministry of Health, State Key Laboratory for Oncogenes and Related Genes, Renji Hospital, School of Medicine Shanghai Jiao Tong University, Shanghai Institute of Digestive Disease Shanghai China; ^7^ CAS Key Laboratory of Quantitative Engineering Biology, Shenzhen Institute of Synthetic Biology, Shenzhen Institute of Advanced Technology, Chinese Academy of Sciences Shenzhen China; ^8^ Division of Gastroenterology, Union Hospital, Tongji Medical College Huazhong University of Science and Technology Wuhan China; ^9^ Department of Laboratory Medicine Medical University Vienna Vienna Austria; ^10^ State Key Laboratory of Cellular Stress Biology, Xiang'an Hospital, School of Life Sciences, Faculty of Medicine and Life Sciences Xiamen University Xiamen China; ^11^ Department of Biostatistics, School of Public Health, Cheeloo College of Medicine, Institute for Medical Dataology Shandong University Jinan China; ^12^ Department of Endocrine and Metabolic Diseases, Shanghai Institute of Endocrine and Metabolic Diseases, Ruijin Hospital Shanghai Jiao Tong University School of Medicine Shanghai China; ^13^ Shanghai National Clinical Research Center for metabolic Diseases, Key Laboratory for Endocrine and Metabolic Diseases of the National Health Commission of the Peoples R China, Shanghai Key Laboratory for Endocrine Tumour, Shanghai Digital Medicine Innovation Center, Ruijin Hospital Shanghai Jiao Tong University School of Medicine Shanghai China; ^14^ MRC Integrative Epidemiology Unit (IEU), Bristol Medical School University of Bristol Bristol United Kingdom; ^15^ Department of Gastroenterology and Hepatology, The Second Affiliated Hospital, School of Medicine South China University of Technology Guangzhou China; ^16^ Guangdong Provincial Key Laboratory of Malignant Tumor Epigenetics and Gene Regulation, Medical Research Center, Sun Yat‐Sen Memorial Hospital Sun Yat‐Sen University Guangzhou China; ^17^ Nanchang Research Institute Sun Yat‐Sen University Nanchang China; ^18^ Department of Clinical Analysis Federal University of Santa Catarina Florianópolis Brazil; ^19^ Department of Gastroenterology and Hepatology, Faculty of Medicine and University Hospital Cologne University of Cologne Cologne Germany; ^20^ Institute of Health and Medicine, Hefei Comprehensive National Science Center Hefei China; ^21^ Department of Gastroenterology and Hepatology, General Hospital, Tianjin Medical University Tianjin Institute of Digestive Diseases, Tianjin Key Laboratory of Digestive Diseases Tianjin China; ^22^ Department of Cardiology The First Affiliated Hospital of Nanjing Medical University, Nanjing Medical University Nanjing China; ^23^ Department of Pathophysiology, Guangdong Provincial Key Laboratory of Proteomics, School of Basic Medical Sciences Southern Medical University Guangzhou China; ^24^ Microbiome Medicine Center, Zhujiang Hospital Southern Medical University Guangzhou China; ^25^ Department of Oncology The First Affiliated Hospital of Anhui Medical University Hefei China; ^26^ Inflammation and Immune Mediated Diseases Laboratory of Anhui Province Anhui Medical University Hefei China; ^27^ Department of Geriatrics, Division of Life Sciences and Medicine, The First Affiliated Hospital of USTC University of Science and Technology of China Hefei China; ^28^ Anhui Key Laboratory of Geriatric Immunology and Nutrition Therapy Hefei China; ^29^ Department of Medicine University of California San Diego La Jolla California USA

## Abstract

Accumulating evidence indicates that the gut microbiota is intricately involved in the initiation and progression of human diseases, forming a multidirectional regulatory axis centered on intestinal microbiota. This article illustrates the challenges in exploring the role of the gut microbiota in inflammatory digestive diseases, such as metabolic dysfunction‐associated steatotic liver disease (MASLD) and inflammatory bowel disease (IBD), and summarizes the existing microbiome‐focused treatment strategies (probiotics, prebiotics, symbiotics, fecal microbiota transplantation, and bacteriophages therapy), emerging technologies (gut microbiome‐on‐a‐chip and artificial intelligence), as well as possible future research directions. Taken together, these therapeutic strategies and technologies present both opportunities and challenges, which require researchers and clinicians to test the rationality and feasibility of various therapeutic modalities in continuous practice.

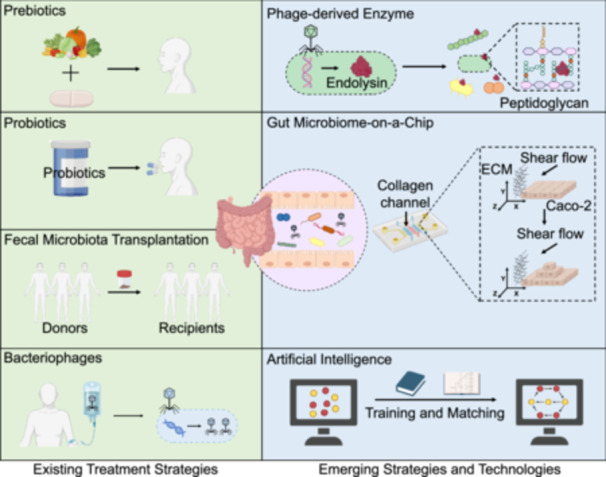

To the Editor,

The gastrointestinal tract accommodates a diverse range of microbes, including bacteria, fungi, viruses, and archaea, which jointly regulate host metabolism, immune responses, redox homeostasis, and disease progression by producing various active substances such as lipopolysaccharide (LPS), trimethylamine, polysaccharides, endogenous alcohol, short‐chain fatty acids (SCFAs), and secondary bile acids [1, 2]. A wealth of preclinical and clinical research has manifested the substantial role of gut microbes and their metabolites in developing and progressing inflammatory digestive diseases. These studies have deepened our comprehension of disease etiology and contributed to developing novel therapeutic strategies targeting the gut microbiota. However, it is important to recognize that research on gut microbes in inflammatory digestive diseases represents both a challenge and an opportunity, with several key issues impeding progress in the field that merit attention.

In this perspective, we illustrated the challenges in exploring the role of the gut microbiota in inflammatory digestive diseases such as metabolic dysfunction‐associated steatotic liver disease (MASLD) and inflammatory bowel disease (IBD), and summarized the existing microbiome‐focused treatment strategies, emerging technologies, as well as possible future research directions.

## CHALLENGES OF GUT MICROBIOME RESEARCH IN INFLAMMATORY DIGESTIVE DISEASES

### Heterogeneity of microbial data

The development of non‐culture techniques for microbiota and multi‐omics sequencing technology has deepened our understanding of how gut microbiota influences health and disease. Numerous studies convincingly show that the gut microbiological profiles of patients differ significantly from those of healthy individuals, with variations spanning all taxonomic levels. A consensus has emerged regarding certain structural characteristics of microbiota at the phylum level. However, at finer taxonomic levels, the gut microbiome compositions of patients exhibit considerable heterogeneity and contradictory findings across studies, presenting a significant challenge in current microbiota and inflammatory digestive disease research. The underlying reasons for this phenomenon are manifold, encompassing factors such as differences in sequencing platforms and regions, statistical biases, variations in the timing of sample collection [3], and fecal microbial load [4], as detailed in Table 1. Collecting a large multi‐center sample cohort will be an effective strategy to address data heterogeneity, and the confounding variables mentioned in Table 1 should also be fully considered in the experimental design. Additionally, establishing minimum quality control standards for microbial experimental design, clinical sample collection, and sequencing analysis must be thoroughly considered to enable effective data integration within a standardized framework.

**Table 1:** Summary of potential causes of heterogeneity in microbial composition of the same disease in different studies.

Classification of causes	Interpretation
Sample bias	※The timing of sample collection can affect the reproducibility of microbiome analyses even more than experimental interventions or dietary changes, and researchers should consider host circadian dynamics in experimental design.
	※The population exhibits considerable individual variability, and the sample size plays a crucial role in influencing the accuracy of the analysis and the reliability of the statistical results.
	※Fecal microbial load is a key factor driving gut microbiome variation and serves as a significant confounder in disease association studies.
Disease stage or subtype identification error	※Disease stage and subtype classification affect the results of microbiological analysis in a population cohort. For example, obese and lean MASLD patients may have significantly different gut microbiota, and mixed analyses may produce misleading results.
Individual factors	※Host genetic background, race, diet, drugs, and other variables can also cause differences in gut microbiota.
Sequencing techniques	※16S rRNA gene sequencing is still the dominant method in current research, but the results are far less detailed and accurate than metagenomic sequencing, which can sequence the entire genome and produce more species information.
	※Primer's choice, reference databases, clustering methods, threshold setting, and specific processes can all cause taxonomic biases.
Sequencing regions	※The 16S rRNA gene sequence contains 10 conserved regions and 9 highly variable regions (V1‐V9), but not every variable region has the same sensitivity. The selection of variable regions has a significant impact on the sequencing results of prokaryotic microbial community structure, with most studies ranging from a single variable region, such as V3 or V4, to two variable regions, such as V3‐V4 or V4‐V5, and some have three variable regions, such as V1‐V3 or V4‐V6.
Sequencing platforms	※The Illumina sequencing platform is widely used in microbiome studies with its lower cost advantages and higher throughput advantages, but the sequences produced by it are short (≤300 bases), and the resolution is limited.
Statistical bias	※*p*‐values are often used to explain whether microbial abundance is statistically significant or not; however, *p*‐values alone do not provide reliable results and require a false discovery rate (FDR) correction, which some studies do not.

Abbreviations: MASLD, metabolic dysfunction‐associated steatotic liver disease; 16S rRNA, 16S ribosomal RNA.

### Incomplete data on microbial‐mediated intestinal metabolomics

Gut microbiota‐derived metabolites are key molecular mediators of microbiome‐host interaction and regulate host immune maturation, immune homeostasis, and mucosal integrity [5]. Advances in mass spectrometry, clinical data, and preclinical studies are illuminating how microbial metabolites contribute to the pathogenesis of inflammatory digestive diseases. However, incomplete metabolomic data remains a major predicament, which is not only reflected in the scarcity of clinical metabolomic data for conditions like primary sclerosing cholangitis (PSC) and acute pancreatitis (AP) but also in the uncertainty of gut metabolite function. For instance, while SCFAs are generally regarded as beneficial in various diseases, Wang et al., recently demonstrated that butyrate and propionate are bacterially derived danger signals that promote interleukin‐1beta (IL‐1β) release through epigenetic regulation by activating the nucleotide‐binding oligomerization domain (NOD), leucine‐rich repeat (LRR), and pyrin domain‐containing protein 3 (NLRP3) in human macrophages [6]. Collecting large transnational cohorts may assist in providing more light on how microbe‐derived metabolites affect disease phenotypes. In addition, innovative methods for detecting unknown compounds, especially metabolites in low concentrations in fecal samples, need to be developed further to advance microbial metabolites from the known to the unknown, breaking the limitation of metabolite data in known knowledge framework studies.

### Insufficient insights into the gut mycobiome and virome in disease

The human gastrointestinal tract harbors a rich diversity of microbes, including bacteria, fungi, viruses, archaea, and protozoa. Currently, the majority of research has focused on alterations in gut bacteria within inflammatory digestive diseases and their influence on disease progression while largely neglecting the role of the gut mycobiome and virome elements, appropriately described as “dark matter” in the context of disease. In reality, intestinal fungi, as eukaryotes, possess genomes about 100 times larger than those of bacteria, display greater biological complexity, and may hold a more significant role in influencing health and disease [7]. Similarly, viruses in the gut, including bacteriophages, act as natural bacterial predators and are instrumental in maintaining the bacterial ecological community [8]. Future research should thus strengthen the study of the role of these two important “dark matters” in disease, particularly emphasizing their evolutionary biology and the development of comprehensive databases.

### Ambiguities in microbial interaction ecological networks

The resource competition and the selection pressure engendered by metabolic activity among microorganisms give rise to diverse ecological interactions not only within bacteria but also between bacteria and fungi or viruses. How these interactions affect the development of inflammatory digestive diseases is a fascinating area. Recent research has revealed that 3‐succinylated cholic acid, a lumen‐restricted bile acid produced by *Bacteroides uniformis*, can alleviate MASLD by promoting the growth of *Akkermansia muciniphila* [9], which exemplifies a typical bacterial cross‐feeding pattern. The reduction or absence of bacterial mutual feeding resources likely contributes to MASLD deterioration, but it is merely one “trade route” among numerous interactions. Therefore, reprogramming the complex ecological networks of bacterial interactions could be pivotal for treating or ameliorating disease, a principle widely applicable to all gut microbiome‐related conditions. Unfortunately, our current comprehension of microbial interactions in diseases remains limited, mostly confined to association analyses without empirical validation, an area that requires substantial reinforcement.

### Limited diagnostic and predictive potency of microbial‐based biomarkers

Identifying reliable and innovative biomarkers is of crucial significance for diagnosing and treating inflammatory digestive diseases. For instance, in contrast to invasive colonoscopy, the current gold standard for diagnosing IBD, biomarker identification could enable early detection and prediction, facilitate timely interventions, and reduce the risk of complications [10]. Nevertheless, the heterogeneity of microbiological data undermines its reliability and reproducibility as a diagnostic biomarker. Encouragingly, recent studies across cohorts have offered promising solutions. Zheng et al., demonstrated that a multibacteria biomarker panel, which included both enriched and depleted species, delivered an excellent diagnostic performance in distinguishing IBD from non‐IBD, as well as Crohn's disease (CD) and ulcerative colitis (UC) across different regions and ethnicities [11]. This method effectively mitigates cohort selection bias and other confounding variables that often hinder cross‐sectional studies. Furthermore, given the relationship between gut microbes, host metabolic status, and genetics, integrating metabolomic and metagenomic data is recommended to identify more robust biomarkers using large‐scale datasets. It is important to emphasize that any novel biomarker should outperform the biomarkers currently in clinical use and maintain robust reproducibility across independent cohorts.

## THERAPEUTIC OPPORTUNITIES AND FUTURE PROSPECTS

**Figure 1:**
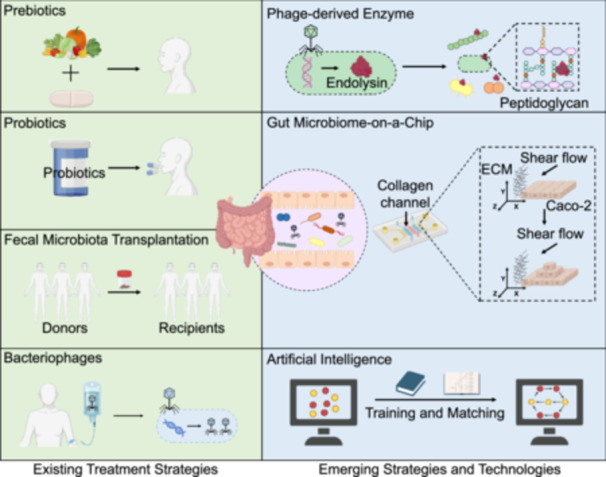
Intervention and therapeutic strategies for inflammatory digestive diseases anchored in gut microbiota. The essence of microbiological therapy lies in augmenting the abundance of beneficial bacteria while diminishing the presence of pathogenic bacteria in the gut (left panel, existing treatment strategies; right panel, Emerging Strategies and Technologies). ECM, extracellular matrix.

Increasing preclinical and clinical studies have indicated that the dysregulation of intestinal microbiota is associated with the development and progression of inflammatory digestive diseases. Manipulating the gut microbiota has emerged as a novel approach for treating gut‐derived diseases, primarily emphasizing replenishing anti‐inflammatory bacteria and their metabolites, eliminating pathogenic bacteria, and restoring the entire gut ecosystem. Current strategies primarily comprise probiotics, prebiotics, symbiotics, fecal microbiota transplantation (FMT), and bacteriophages (Figure 1). For instance, a recent randomized clinical trial revealed that FMT could significantly alleviate the disease phenotype of patients with nonalcoholic fatty liver disease (NAFLD, MASLD's former name) by improving the imbalance of intestinal microbiota, and its clinical efficacy was higher in lean NAFLD than in obese NAFLD patients [12]. This finding underscores the potential of FMT as a therapeutic strategy for NAFLD and suggests that its effectiveness may vary depending on disease subtypes. However, it is significant to note that current research on FMT is still limited, with only a few blinded randomized controlled trials. This is largely attributed to the broad and nonspecific nature of FMT, which provides scant information on long‐term effects on individuals and raises concerns regarding its safety. When using FMT to treat diseases, the precise matching of donor and recipient should be strictly implemented, similar to blood type and organ matching, to ensure the most beneficial outcome. Additionally, the efficacy of remodeling gut microbiota homeostasis in treating disease varies among individuals and may be influenced by the properties of baseline microbiota, the abundance of opportunistic pathogens, the degree of functional redundancy, and the stage of the disease [13]. Colonization resistance, where the natural intestinal microbiota colonizes host tissue to exclude potential pathogen infection (whether resident or invasive), is also a crucial factor influencing the remodeling of the gut microbiota [14].

In recent years, utilizing bacteriophages that specifically target pathogenic bacteria has attracted extensive attention as a precision medicine approach. As natural predators of bacteria, phages thrive in environments where bacteria exist. Viral particles vastly outnumber bacteria in the gut, with over 90% being phages. Intestinal phages of healthy adults are predominantly individual and highly diverse, with crAss‐like and Microviridae phages being the most stable colonizers, which may influence human health by affecting gut microbiota [15]. Cornuault JK et al., discovered that *Faecalibacterium prausnitzii* was depleted in IBD patients compared to healthy individuals, while the abundance of mild phages targeting these bacteria was significantly higher in the stool samples [16]. This endogenous targeting offers a new avenue for microbiota‐mediated disease treatment in the “post‐antibiotic era,” as phages have a narrow host range and can specifically reduce the levels of certain pathogenic bacteria without disturbing the surrounding microbiota.

Within this theoretical framework, researchers have developed phage mixtures for common inflammatory digestive diseases such as IBD, alcoholic liver disease (ALD), MASLD, and PSC, based on the dominant pathogen characteristics of patients [17]. These mixtures have shown promising efficacy in animal studies and small human cohorts. However, it is important to recognize that phage therapy is still in its early stages. In addition to the absence of comprehensive laws and regulations, several issues within the theoretical framework need to be addressed, such as phage dose determination, bacterial lysis‐induced localized inflammation, and understanding the pharmacokinetic and pharmacodynamic properties. More fundamental and preclinical studies and well‐designed randomized, blinded, placebo‐controlled clinical trials are requisite to advance the field. Furthermore, the resurgence of phage therapy has stimulated the development of phage‐related therapeutic strategies, and phage endolysin is regarded as a novel drug candidate with great potential. Phage endolysins are cell wall hydrolases encoded by bacteriophages during their late replication stages. These enzymes target peptidoglycan in the bacterial cell wall, leading to cell wall rupture and bacterial death. Recent studies have indicated that phage endolysins not only precisely target intestinal pathogens but also effectively eradicate bacterial biofilms, which can effectively address the dilemma that antibiotics have difficulty eliminating disease‐causing bacteria biofilms [18]. Therefore, the development of phage endolysins using computational biology, bioinformatics, and synthetic biology may usher in a new dawn for disease treatment.

Technological innovations and breakthroughs have also brought forth new horizons for diagnosing and treating inflammatory digestive diseases (Figure 1). For instance, Lee et al., recently proposed a scalable gut microbiome‐on‐a‐chip (GMoC) with a reproducible 3D stratified gut epithelium derived from Caco‐2 cells, which can visualize the behavior and interactions of gut microbiota and their collective influence on gut health and disease through high‐magnification imaging [19]. This novel technique offers an efficient and effective biomimetic scaffold for cultivating gut microbes and studying their effects on the gut to discover new targets in the mechanisms by which microbes induce disease and facilitate the development of strategies for effective microbial therapies. Furthermore, artificial intelligence (AI), driven by cutting‐edge algorithms such as machine learning, deep learning models, neural networks, deep generative models, graphical processing units, and interpretable models, is revolutionizing the diagnosis and treatment of diseases [20]. The human microbiome is an ecosystem characterized by highly dynamic and complex microbe–microbe, host–microbe, and microbe–environment interactions. Integrating multiple AI models can efficiently manage and interpret large datasets, including multi‐omics sequencing data, and enable the identification and analysis of temporal and spatial interactions within the gut ecological network. This facilitates the early diagnosis of disease, prediction of treatment response, and monitoring of disease activity, progression, and recurrence. However, it is crucial to note that the clinical application of AI still confronts several challenges, including issues related to data quality, reproducibility, universality, and the ethical, legal, and regulatory concerns surrounding its use. As AI continues to evolve at the intersection of research and clinical practice, it is imperative that rigorous trials be conducted to evaluate its clinical efficacy and ensure its benefits.

## CONCLUSION

Exploring the niche changes of gut microbiota provides novel insights into the understanding of human inflammatory digestive diseases. Nevertheless, the heterogeneity of microbial data, influenced by numerous confounding variables, remains a ubiquitous challenge in population cohort studies. Collecting large multicenter clinical cohorts may assist in solving this predicament, but for some rare diseases like PSC, relying on this advantage of quantity becomes difficult. Meanwhile, establishing minimum quality control standards for the design of microbial experiments and sample collection within population cohorts, alongside strengthening the application of integrated multi‐omics analysis in microbial studies, can better discover combined disease biomarkers and elucidate the mechanisms underlying microbial interaction in disease regulation. Additionally, microbial‐based therapeutic strategies and technologies present both opportunities and challenges, which require researchers and clinicians to test the rationality and feasibility of various therapeutic modalities in continuous practice.

## AUTHOR CONTRIBUTIONS

**Yongpeng Shi**: Writing—original draft; writing—review and editing. **Zeran Chen**: Writing—original draft; writing—review and editing. **Tingyu Fang**: Writing—original draft; writing—review and editing. **Xingyao Chen**: Writing—original draft. **Youpeng Deng**: Writing—original draft. **Hao Qin**: Writing—original draft. **Min Lian**: Writing—original draft. **Juntao Shen**: Writing—original draft. **Yuru Zong**: Writing—original draft. **Huikuan Chu**: Writing—original draft. **Constanze Hoebinger**: Writing—original draft. **Hao Guo**: Writing—review and editing. **Zhongshang Yuan**: Writing—review and editing. **Jie Zheng**: Writing—review and editing. **Yongjian Zhou**: Writing—review and editing. **Yue Pan**: Writing—review and editing. **Beatriz G Mendes**: Writing—review and editing. **Sonja Lang**: Writing—review and editing. **Tim Hendrikx**: Writing—review and editing. **Suling Zeng**: Writing—review and editing. **Hailong Cao**: Writing—review and editing. **Ling Yang**: Writing—review and editing. **Lianmin Chen**: Writing—review and editing. **Peng Chen**: Writing—review and editing. **Lei Dai**: Writing—review and editing. **Hua Wang**: Writing—review and editing. **Shi Yin**: Writing—review and editing. **Shu Zhu**: Writing—review and editing. **Xiong Ma**: Writing—review and editing. **Bernd Schnabl**: Writing—review and editing. **Hanqing Chen**: Project administration; conceptualization; supervision; writing—review and editing; funding acquisition. **Yi Duan**: Conceptualization; project administration; writing—review and editing; supervision; funding acquisition.

## CONFLICT OF INTEREST STATEMENT

Bernd Schnabl has been consulting for Ambys Medicines, Ferring Research Institute, Gelesis, HOST Therabiomics, Intercept Pharmaceuticals, Mabwell Therapeutics, Patara Pharmaceuticals, Surrozen and Takeda. Bernd Schnabl's institution, UC San Diego, has received research support from Axial Biotherapeutics, BiomX, ChromoLogic, CymaBay Therapeutics, Intercept, NGM Biopharmaceuticals, Prodigy Biotech and Synlogic Operating Company. Bernd Schnabl is founder of Nterica Bio. UC San Diego has filed several patents with Yi Duan, Sonja Lang, and Bernd Schnabl as inventors related to this work. The other authors declare no conflict of interest.

## ETHICS STATEMENT

No animals or humans were involved in this study.

## Data Availability

Data sharing is not applicable to this article as no new data were created or analyzed in this study. This paper does not generate any new data. Supplementary materials (graphical abstract, slides, videos, Chinese translated version, and updated materials) may be found in the online DOI or iMeta Science http://www.imeta.science/.
